# Associations of health literacy with dental care use and oral health status in Japan

**DOI:** 10.1186/s12889-023-15866-7

**Published:** 2023-06-05

**Authors:** Keiko Murakami, Jun Aida, Shinichi Kuriyama, Hideki Hashimoto

**Affiliations:** 1 Tohoku Medical Megabank Organization, Tohoku University, 2-1 Seiryo-machi, Aoba-ku, 980-8573 Sendai, Miyagi Japan; 2 Department of Molecular Epidemiology, Tohoku University Graduate School of Medicine, Sendai, Miyagi Japan; 3 Department of Health and Social Behavior, School of Public Health, The University of Tokyo, Bunkyo-ku, Tokyo, Japan; 4 Department of Oral Health Promotion, Graduate School of Medical and Dental Sciences, Tokyo Medical and Dental University, Bunkyo-ku, Tokyo, Japan; 5 Department of Disaster Public Health, International Research Institute of Disaster Science, Tohoku University, Sendai, Miyagi Japan

**Keywords:** Curative dental care use, Health literacy, Japan, Oral health, Preventive dental care use

## Abstract

**Background:**

The concept of health literacy has gained prominence in the context of oral health. In Japan, curative dental care is generally under universal health coverage, while preventive dental care requires effort. We used this situation to test the hypothesis that high health literacy is associated with preventive dental care use and good oral health status, but not with curative dental care use, in Japan.

**Methods:**

A questionnaire survey was conducted from 2010 to 2011 among residents aged 25–50 years in Japanese metropolitan areas. Data from 3767 participants were used. Health literacy was measured using the Communicative and Critical Health Literacy Scale, and the total score was categorized into quartiles. Poisson regression analyses with robust variance estimators were conducted to examine the associations of health literacy with curative dental care use, preventive dental care use, and good oral health, adjusted for covariates.

**Results:**

The percentages of curative dental care use, preventive dental care use, and good oral health were 40.2%, 28.8%, and 74.0%, respectively. Health literacy was not associated with curative dental care use; the prevalence ratio (PR) of the highest relative to the lowest quartile of health literacy was 1.04 (95% confidence interval [CI], 0.93–1.18). High health literacy was associated with preventive dental care use and good oral health; the corresponding PRs were 1.17 (95% CI, 1.00–1.36) and 1.09 (95% CI, 1.03–1.15), respectively.

**Conclusions:**

These findings may provide clues for the design of effective interventions to promote preventive dental care use and improve oral health status.

## Background

Health literacy, which is defined as people’s knowledge, motivation, and competence to access, understand, appraise, and apply health information to make judgments and take decisions in everyday life regarding healthcare, disease prevention, and health promotion to maintain or improve their quality of life during the life course [1], is an important factor for health behaviors and health status [2]. Studies have reported that people with low health literacy cannot adequately understand and utilize information regarding health in a way that protects and improves their health, and therefore they may have lower health status [3].

The concept of health literacy has gained prominence in the context of oral health, which is known as oral health literacy [4]. A systematic review on the association between oral health literacy and oral conditions presented inconclusive data on this association [5]. A meta-analysis further indicated a lack of association between oral health literacy and frequency of visit to the dentist [6]. There seem to be two main problems with the previous studies. The first is that most of the studies were conducted on non-representative samples of care-seeking populations, such as patients attending dental clinics [5, 6]. Because these populations may have different profiles to the general population, studies carried out on populations within the general population (e.g., outside the health system) are necessary to provide clear conclusions. The second is that the majority of the studies examining the association between oral health literacy and dental care use did not differentiate the reasons for the visits (preventive vs. problem-based) [6, 7]. Furthermore, general health literacy has rarely been examined in the context of oral health. As oral health is part of general health, health literacy research should include oral health-related actions and decisions.

Health literacy is considered to have three levels: functional, communicative, and critical [8]. Functional health literacy describes basic-level skills that are sufficient for individuals to obtain relevant health information and to apply that knowledge to a range of prescribed activities [8]. Communicative health literacy refers to the capacity to collect necessary and appropriate information to support one’s actions and communicate the information to others, and critical health literacy refers to one’s capacity to critically evaluate the quality of available information and select appropriate information for use in decision-making [8]. Recently, communicative and critical health literacy has been increasingly acknowledged to enable effective health-protective behaviours and facilitate the achievement of subsequent health outcomes among Japanese adults [9, 10], although Japanese adults display the highest levels of proficiency in literacy and numeracy among adults in the OECD countries [11]. However, the majority of the studies on health literacy in the oral health domain have solely focused on functional health literacy, including word recognition, reading comprehension, and computation [12].

Japan achieved universal health coverage in 1961 [13] and had the highest dental care use with relatively low out-of-pocket dental expenditures [14]. Health Japan 21 (the second term) aims to increase the percentage participating in annual dental check-ups from 34.1% in 2009 to 65% in 2022 and to decrease the percentage of individuals in age 40s with progressive periodontitis from 37.3% in 2005 to 25% in 2022 [15]. In the current national context in Japan, curative treatments are the major type of care provided in dental clinics under the universal health care insurance, while preventive dental care use requires effort because it is less frequently covered by the universal health care insurance and less urgent [14]. A study in Japan found that the price elasticity for dental check-ups visits was higher than for dental treatment visits [16]. To the best of our knowledge, however, no studies have examined the associations of health literacy with curative dental care use and preventive dental care use in Japan. We aimed to test the hypothesis that high health literacy is associated with preventive dental care use and good oral health status, but is not associated with curative dental care use, among community-dwelling adults in Japan.

## Methods

### Study population

The present study was derived from the Japanese Study of Stratification, Health, Income, and Neighborhood (J-SHINE), the details of which were described elsewhere [17–20]. The aim of the J-SHINE is to clarify the complex associations between social factors and health from interdisciplinary perspectives [17]. From October 2010 to February 2011, the J-SHINE survey was carried out in four municipalities in and around the greater Tokyo metropolitan area [17]. Of 13,920 residents aged 25–50 years who were probabilistically selected from the residential registry, survey staff members were able to contact 8408 residents [17]. The participants were asked to complete a computer-assisted questionnaire unless they requested a face-to-face interview, and 4317 participants gave valid responses [17]. We analysed 3767 participants without missing values for any variables examined in the present study. The Research Ethics Committee of The University of Tokyo, Graduate School of Medicine approved the survey procedure of the J-SHINE. The J-SHINE Data Management Committee approved the authors’ secondary use of the data, with personally identifiable information deleted to ensure confidentiality.

### Measures

Health literacy was measured using the Communicative and Critical Health Literacy (CCHL) Scale [10], which was developed and validated in Japan to assess communicative and critical health literacy [8]. The scale includes three items for communicative health literacy (items 1–3) and two items for critical health literacy (items 4–5). The participants were asked whether they could do the following: (1) collect health-related information from various sources; (2) extract the information they want; (3) understand and communicate the obtained information; (4) consider the credibility of the information; and (5) make decisions based on the information, in the context of health issues [10]. Each item was rated on a five-point Likert scale, ranging from 1 (strongly disagree) to 5 (strongly agree). The scores of the five responses were summed and divided by the number of items to determine a total score (theoretical range: 1–5), with higher scores indicating greater health literacy. The Cronbach’s alpha value for the scale was 0.84. The total score was categorized into quartiles.

Curative dental care use in the past year was measured with the question, “Have you been seen by a dentist or a dental hygienist in the past year? Exclude use for dental scaling and fluoride and orthodontic treatments” [18, 20]. Preventive dental care use in the past year was measured with the question, “Have you been seen by a dentist or a dental hygienist for dental scaling or fluoride or orthodontic treatments in the past year?” [18, 20]. Oral health was measured with the question “How would you describe the health of your teeth and gums? Would you say it is excellent, very good, good, fair, or poor?” Responses were dichotomized as good oral health (excellent, very good, or good) and poor oral health (fair or poor) [18–20].

We selected the following covariates: age (25–34, 35–44, 45–50 years), sex, marital status (married, unmarried [single, divorced, widowed]), working status (working, not working), educational attainment, and equivalent household income [18–22]. Educational attainment was divided into three categories: high school or lower (elementary, junior high school, senior high school), college (2-year college, special training school), and university or higher (university, graduate school). Participants selected their total annual household income from 15 response categories. Equivalent household income was calculated as household income adjusted for household size, using the OECD-modified equivalence scale [23]. We used individual income as equivalent household income for participants whose household income was unknown or missing but who responded on individual income.

### Statistical analysis

Poisson regression analyses with robust variance estimators were conducted to examine the associations of health literacy with curative dental care use, preventive dental care use, and oral health status, because the prevalences of outcomes were high (> 10%) and logistic regression analysis would overestimate the effect [24]. Prevalence ratios (PRs) and their 95% confidence intervals (CIs) were calculated with adjustment for age, sex, marital status, working status, educational attainment, and equivalent household income. The lowest level of health literacy was set as the reference category.

All analyses were conducted using Stata 16.0 (StataCorp LP, College Station, TX, USA), and a two-tailed *P* < 0.05 was considered statistically significant.

## Results

Table 1 shows the characteristics of the participants. Among the participants, 19.9% were 45–50 years old, 52.9% were women, 70.5% were married, 80.0% were working, and 44.5% had graduated from university or higher. The mean of equivalent household income was 3631.0 thousand Japanese yen. The mean score for health literacy was 3.63 (standard deviation, 0.64). The percentage of participants with curative dental care use in the past year was 40.2%, while the percentage of participants with preventive dental care use in the past year was 28.8%. Among the participants, 74.0% self-reported that their oral health was excellent, very good, or good.

**Table 1:** Characteristics of the participants from the Japanese Study of Stratification, Health, Income, and Neighborhood (J-SHINE) (n = 3767)

	n (%) / mean (SD)	
Age, n (%)		
25–34 years	1396	(37.1)
35–44 years	1620	(43.0)
45–50 years	751	(19.9)
Women, n (%)	1992	(52.9)
Married, n (%)	2655	(70.5)
Working, n (%)	3013	(80.0)
Educational attainment, n (%)		
High school or lower	834	(22.1)
College	1257	(33.4)
University or higher	1676	(44.5)
Equivalent household income^a^, mean (SD)	3631.0	(2192.4)
Health literacy, mean (SD)	3.63	(0.64)
Curative dental care use, n (%)	1515	(40.2)
Preventive dental care use, n (%)	1083	(28.8)
Good oral health, n (%)	2786	(74.0)

SD, standard deviation.

^a^Thousand Japanese yen (/year)

Table 2 presents the PRs and 95% CIs for curative dental care use. Health literacy was not associated with curative dental care use; the crude PRs of the second, third, and fourth quartiles relative to the first quartile of health literacy were 1.06 (95% CI, 0.96–1.17), 0.93 (95% CI, 0.82–1.05), and 1.05 (95% CI, 0.93–1.19), respectively. No association remained after adjustment for covariates; the corresponding multivariate-adjusted PRs were 1.05 (95% CI, 0.95–1.16), 0.90 (95% CI, 0.80–1.02), and 1.04 (95% CI, 0.93–1.18), respectively. Older age and marital status were associated with curative dental care use, while sex, working status, educational attainment, and equivalent household income were not.

**Table 2:** PRs and 95% CIs for curative dental care use

	% of curative dental care use	Crude			Multivariate-adjusted^a^		
		PR (95% CI)			PR (95% CI)		
Health literacy							
1st quartile (lowest)	39.6	1.00			1.00		
2nd quartile	42.0	1.06	(0.96–1.17)		1.05	(0.95–1.16)	
3rd quartile	36.8	0.93	(0.82–1.05)		0.90	(0.80–1.02)	
4th quartile (highest)	41.6	1.05	(0.93–1.19)		1.04	(0.93–1.18)	
Age							
25–34 years	39.3	1.00			1.00		
35–44 years	38.3	0.97	(0.89–1.07)		0.94	(0.85–1.03)	
45–50 years	46.2	1.18	(1.06–1.30)		1.12	(1.00–1.24)	
Sex							
Men	38.7	1.00			1.00		
Women	41.6	1.08	(1.00–1.16)		1.08	(1.00–1.18)	
Marital status							
Married	42.1	1.00			1.00		
Unmarried	35.8	0.85	(0.78–0.93)		0.85	(0.77–0.94)	
Working status							
Working	40.3	1.00			1.00		
Not working	39.8	0.99	(0.89–1.09)		0.94	(0.84–1.04)	
Educational attainment							
High school or lower	40.3	1.00			1.00		
College	41.1	1.02	(0.92–1.13)		1.02	(0.92–1.13)	
University or higher	39.5	0.98	(0.89–1.09)		1.00	(0.90–1.11)	
Equivalent household income							
1st quartile (lowest)	39.4	1.00			1.00		
2nd quartile	39.6	1.00	(0.90–1.12)		0.99	(0.89–1.10)	
3rd quartile	40.1	1.02	(0.91–1.14)		1.02	(0.91–1.15)	
4th quartile (highest)	42.0	1.07	(0.96–1.19)		1.05	(0.93–1.18)	

PR, prevalence ratio; 95% CI, 95% confidence interval

^a^Adjusted for all other variables in the table.

Table 3 presents the PRs and 95% CIs for preventive dental care use. High health literacy was associated with preventive dental care use; the crude PRs of the second, third, and fourth quartiles relative to the first quartile of health literacy were 1.05 (95% CI, 0.92–1.20), 1.02 (95% CI, 0.88–1.19), and 1.21 (95% CI, 1.04–1.40), respectively. These associations did not materially change after adjustment for covariates; the corresponding multivariate-adjusted PRs were 1.02 (95% CI, 0.89–1.16), 0.95 (95% CI, 0.81–1.11), and 1.17 (95% CI, 1.00–1.36), respectively. Older age, women, higher level of education, and higher income were associated with preventive dental care use, while marital status and working status were not.

**Table 3:** PRs and 95% CIs for preventive dental care use

	% of preventive dental care use	Crude			Multivariate-adjusted^a^		
		PR (95% CI)			PR (95% CI)		
Health literacy							
1st quartile (lowest)	27.2	1.00			1.00		
2nd quartile	28.6	1.05	(0.92–1.20)		1.02	(0.89–1.16)	
3rd quartile	27.7	1.02	(0.88–1.19)		0.95	(0.81–1.11)	
4th quartile (highest)	32.8	1.21	(1.04–1.40)		1.17	(1.00–1.36)	
Age							
25–34 years	27.9	1.00			1.00		
35–44 years	27.4	0.98	(0.88–1.10)		0.98	(0.87–1.11)	
45–50 years	33.3	1.19	(1.05–1.36)		1.16	(1.01–1.33)	
Sex							
Men	23.6	1.00			1.00		
Women	33.3	1.41	(1.27–1.57)		1.42	(1.27–1.59)	
Marital status							
Married	29.6	1.00			1.00		
Unmarried	26.8	0.91	(0.81–1.02)		0.97	(0.86–1.10)	
Working status							
Working	27.6	1.00			1.00		
Not working	33.4	1.21	(1.08–1.36)		1.10	(0.97–1.25)	
Educational attainment							
High school or lower	24.1	1.00			1.00		
College	30.9	1.28	(1.11–1.48)		1.18	(1.02–1.37)	
University or higher	29.5	1.22	(1.06–1.41)		1.18	(1.02–1.37)	
Equivalent household income							
1st quartile (lowest)	23.6	1.00			1.00		
2nd quartile	27.8	1.18	(1.01–1.37)		1.19	(1.03–1.39)	
3rd quartile	29.9	1.27	(1.09–1.48)		1.30	(1.11–1.52)	
4th quartile (highest)	34.3	1.46	(1.26–1.68)		1.46	(1.25–1.71)	

PR, prevalence ratio; 95% CI, 95% confidence interval

^a^Adjusted for all other variables in the table.

Table 4 presents the PRs and 95% CIs for good oral health. High health literacy was associated with good oral health; the crude PRs of the second, third, and fourth quartiles relative to the first quartile of health literacy were 1.03 (95% CI, 0.97–1.08), 1.09 (95% CI, 1.03–1.15), and 1.11 (95% CI, 1.05–1.17), respectively. These associations did not materially change after adjustment for covariates; the corresponding multivariate-adjusted PRs were 1.01 (95% CI, 0.96–1.07), 1.06 (95% CI, 1.01–1.13), and 1.09 (95% CI, 1.03–1.15), respectively. Younger age, women, higher level of education, and higher income were associated with good oral health, while marital status and working status were not.

**Table 4:** PRs and 95% CIs for good oral health

	% of good oral health	Crude			Multivariate-adjusted^a^		
		PR (95% CI)			PR (95% CI)		
Health literacy							
1st quartile (lowest)	70.7	1.00			1.00		
2nd quartile	72.5	1.03	(0.97–1.08)		1.01	(0.96–1.07)	
3rd quartile	76.9	1.09	(1.03–1.15)		1.06	(1.01–1.13)	
4th quartile (highest)	78.4	1.11	(1.05–1.17)		1.09	(1.03–1.15)	
Age							
25–34 years	75.6	1.00			1.00		
35–44 years	74.9	0.99	(0.95–1.03)		1.00	(0.96–1.04)	
45–50 years	68.8	0.91	(0.86–0.96)		0.91	(0.85–0.96)	
Sex							
Men	70.5	1.00			1.00		
Women	77.0	1.09	(1.05–1.13)		1.11	(1.07–1.16)	
Marital status							
Married	73.8	1.00			1.00		
Unmarried	74.5	1.01	(0.97–1.05)		1.00	(0.96–1.05)	
Working status							
Working	73.6	1.00			1.00		
Not working	75.5	1.03	(0.98–1.07)		0.99	(0.95–1.05)	
Educational attainment							
High school or lower	64.8	1.00			1.00		
College	75.3	1.16	(1.10–1.23)		1.13	(1.06–1.20)	
University or higher	77.6	1.20	(1.13–1.27)		1.17	(1.10–1.24)	
Equivalent household income							
1st quartile (lowest)	68.6	1.00			1.00		
2nd quartile	73.8	1.08	(1.02–1.14)		1.07	(1.01–1.13)	
3rd quartile	75.0	1.09	(1.03–1.16)		1.08	(1.01–1.14)	
4th quartile (highest)	78.8	1.15	(1.09–1.21)		1.13	(1.07–1.20)	

PR, prevalence ratio; 95% CI, 95% confidence interval

^a^Adjusted for all other variables in the table.

## Discussion

The present study examined the associations of communicative/critical health literacy with curative dental care use, preventive dental care use, and oral health status among community-dwelling Japanese adults. Health literacy was not associated with curative dental care use, while participants with high level of health literacy were more likely to use preventive dental care than participants with low health literacy. Participants with high level of health literacy were more likely to report good self-perception of oral health than participants with low level of health literacy.

Scores for health literacy were not associated with curative dental care use. Studies in the United States showed no association between oral health literacy and use of any type of dental care [6, 7]. Studies in Brazil showed that low level of oral health literacy and low level of general health literacy were associated with emergency dental care use [25, 26]. It is not possible to compare these findings with the present findings, because there are differences in the dental care policies and potential cultural differences related to the norms and values of oral health across countries. In Japan, the universal public health insurance has mainly covered curative dental treatments since almost the entire general population became insured in 1961 and very few people take out private dental insurance [13]. The universal public health insurance covers a wide range of dental treatments, and the availability of these treatments at low cost enables people in Japan to visit dentists more frequently than people in other OECD countries [14]. As the universal public health insurance mainly covers curative treatments rather than preventive treatments, dental care use in Japan has traditionally been treatment-oriented [18]. In the J-SHINE survey, we previously found that people who self-assessed their oral health to be poor were more likely to use curative dental care than other people, while social variables such as education, income, and social relationships were not associated with curative dental care use [18, 20]. The present study also found that age and marital status were associated with curative dental care use. Taken together, demographic variables and perceived needs, rather than psychosocial factors, appear to be important determinants for curative dental care use in Japan.

High scores for health literacy were associated with preventive dental care use. There is limited and inconclusive evidence on the association between health literacy and preventive dental care use. A study in the United States showed no association between oral health conceptual knowledge and undergoing dental cleaning in the past year [27]. A study in Japan found that communicative/critical health literacy measured by the CCHL scale was not associated with dental check-ups in the past year among Japanese residents who registered with a commercial survey research company database [28]. However, another study found that people with high level of functional oral health literacy (the degree of dental knowledge) were more likely to have regular dental check-ups among community-dwelling adults in a rural area in Japan [29]. We previously reported that older age, higher level of education, and higher income were associated with preventive dental care use in the J-SHINE survey [18]. The present study also found these associations. However, the association between communicative/critical health literacy and preventive care use remained even after adjustment for these variables. The observed association may be partly due to relatively low out-of-pocket dental expenditures in Japan [14]. The provision of preventive dental care was reported to be relatively limited, although some forms of preventive dental care use such as dental scaling are covered by the universal public health insurance if patients have minor dental trouble in Japan [30, 31]. The concept of preventive dental care has become widespread in recent decades, and there have been improvements in oral health behaviours in Japan [32]. However, the spreading of preventive behaviours is often accompanied by inequalities, arising from differential uptake of new information and skills. One comparative study suggested that health literacy in the Japanese general population is lower than that in the general populations of European countries, partly because Japanese respondents found it more difficult to judge and apply health information, suggesting the presence of difficulties in health decision-making in Japan [33]. Communicative health literacy is defined as more advanced-level skills that enable people to extract information regarding health and derive meaning from several forms of communication, to apply new information to changing circumstances, and to interact with others to extend the information and make decisions [8]. Critical health literacy is defined as the most advanced-level skills that allow people to critically analyze information from a variety of sources and information regarding health determinants, and to use this information to exert greater control over situations that affect health [8]. People with high level of communicative/critical health literacy would find it easier to judge and apply information on oral health including preventive dental care.

High scores for health literacy were associated with good self-perception of oral health. The existing scientific evidence is weak or unsubstantiated regarding the associations between oral health literacy and oral health outcomes [5]. However, the majority of the previous studies measured functional oral health literacy mainly using the Rapid Estimate of Adult Literacy in Dentistry (REALD)-30 [34], in clinical settings [5]. These instruments are intended for English-speaking participants, and may not be directly applicable to Japanese-speaking participants. One study targeting community-dwelling adults in a rural area in Japan developed an oral health literacy test to measure the ability of Japanese individuals to recognize basic terms and knowledge specifically related to dentistry, that is functional health literacy, and found that higher oral health literacy was associated with good clinical oral health status, defined as the number of natural teeth, number of decayed teeth, number of functional tooth units, and the community periodontal index [29]. Our finding is consistent with this result. We previously reported that younger age, higher level of education, and higher income were associated with good self-perception of oral health in the J-SHINE survey [19]. The present study also found these associations. However, the association between communicative/critical health literacy and self-perception of oral health remained even after adjustment for these variables. There is only limited evidence on the association between communicative/critical health literacy and oral health status. A study in Brazil that adopted the CCHL scale for oral health found that low health literacy was associated with the presence of dental plaque [25]. These findings suggest that high level of communicative/critical health literacy is associated with good oral health in population-based studies.

The present findings have implications for oral health policy. Japan has achieved one of the lowest out-of-pocket dental expenditure rates and highest dental care use rates globally [14]. However, the present findings that high scores for communicative/critical health literacy were associated wdith preventive dental care use and good oral health suggest that improvements in these levels of health literacy could lead to more frequent use of preventive dental care and improvement of oral health status. Only few interventions to improve health literacy in community settings have incorporated the concept of critical health literacy [35, 36]. The CCHL scale attempts to assess critical health literacy in terms of information appraisal and to ask people the extent to which they consider the reliability, validity, credibility, and applicability of information regarding health [10, 37]. One study showed that health literacy measured by the CCHL scale was significantly improved after an educational program in a community setting [38]. The concept of health literacy is attractive because it fits with the concept of health promotion, and it is considered a personal ‘asset’ that can be developed through educational and other interventions to support greater individual and community control over a variety of health determinants [35]. Interventions that actively use the concept of communicative/critical health literacy in their design would be effective for improving oral health.

Limitations of the present study should be noted. First, because this was a cross-sectional study, the causal direction of the observed associations could not be determined and the dynamic nature of the constructs could not be assessed. It is undeniable that dental care use can improve health literacy. Second, the J-SHINE survey was conducted in urban areas, where the demographic structure and access to dental care may differ from those in rural areas. Third, the CCHL scale may not cover the entire concept of communicative/critical health literacy as defined by Nutbeam [8, 10], although it is a validated questionnaire. Fourth, oral health status was based on self-reports. The inclusion of objective measures should be considered in future studies. Finally, the definitions of dental care can differ among studies and countries [39, 40]. The present study examined curative dental care use and preventive dental care use separately, which is a strength. We believe that the definition of preventive dental care in the present study is suitable at the current time, because dental scaling, fluoride, and orthodontic treatments are a better proxy for placing a high priority on good oral health than other dental care treatments such as fillings, crowns, and root canals.

In conclusion, the present study found that people with high levels of communicative/critical health literacy were more likely to use preventive dental care and have good oral health, while there was no association between communicative/critical health literacy and curative dental care use among community-dwelling adults in Japan. These findings indicate that consideration of health literacy could be useful for public health interventions to promote preventive dental care use and improve oral health status.

## Data Availability

The datasets used during the current study are available from the corresponding author on reasonable request.

## Abbreviations

CCHL: Communicative and Critical Health Literacy
J-SHINE: Japanese Study of Stratification, Health, Income, and Neighborhood
PR: prevalence ratio
95% CI: 95% confidence interval
